# Temporal changes in left ventricular function and paravalvular aortic regurgitation after transcatheter aortic valve implantation: a cardiac magnetic resonance imaging study

**DOI:** 10.1186/1532-429X-14-S1-P94

**Published:** 2012-02-01

**Authors:** Constanze Merten, Hans-Wilko Beurich, Dirk Zachow, Ahmad E Mostafa, Ken Gordian, Ralph Toelg, Volker Geist, Mohamed Abdel-Wahab, Gert H Richardt

**Affiliations:** 1 Cardiology, Heart Center , Segeberger Kliniken, Bad Segeberg, Germany; 2 Radiology, Segeberger Kliniken, Bad Segeberg, Germany

## Background

Transcatheter aortic valve implantation (TAVI) has been established as a therapeutic alternative in patients with severe aortic stenosis at high risk for surgery.

Paravalvular aortic regurgitation (AR) is frequent after TAVI. Yet, little data is available about temporal changes of AR and the impact of TAVI on left ventricular (LV) function and dimensions.

We used cardiac MRI to evaluate LV function, volumes and mass, the occurrence and degree of AR in the early and medium-term follow-up after TAVI.

## Methods

In 21 patients who underwent transfemoral TAVI we performed baseline cardiac MRI at a median of 21 ± 12 days after TAVI and follow-up MRI 10.7 ± 7.2 months later.

LV volumes and function were assessed using standard cine MRI sequences; phase contrast imaging was conducted to measure aortic regurgitation. A calculated regurgitation fraction (RF) of ≥1%-15% was graded as mild, 16%-30% was graded as moderate, 31%-50% as moderate to severe and > 50% was graded severe, a RF <1% was classified as no AR.

## Results

57% of the 21 patients were women. The median age of the patients was 81 years with a median logistic EuroScore of 24.3%.

At baseline MRI, the median LV ejection fraction was 58.1% (range 22.1 to 71.7%) and improved significantly at follow-up to 64.2 % (range 24.0 to 73.5%), p=0.0002.

We observed a significant reduction of the median enddiastolic volume (139.7 ml vs. 122.6 ml, p=0.03) and of LV mass (153.2 ± 32.4 vs. 140.5 ± 37.2 g, p=0.02) from baseline to follow-up MRI.

At baseline MRI, aortic RF was mild in 13 patients and moderate in 4 patients. At follow-up any grade of AR was present in all patients with AR being mild in 16 patients, moderate in 3 patients and moderate to severe in further 3 patients. Over time, the median aortic RF increased slightly but significantly (p=0.02) from 8.0% (range 0.1 to 27.2%) to 8.1% (range 1.7 to 41.9%).

## Conclusions

At mid-term follow-up, cardiac MRI revealed AR, that was mostly mild, in all patients who underwent TAVI. From baseline to follow-up we observed a slight but significant increase in AR. We also could demonstrate a significant improvement of systolic LV function as well as a reduction of LV volumes and mass at mid-term follow-up after TAVI.

## Funding

None.

